# 200. Comparison of Clinical Outcomes among Patients Treated with Ceftriaxone 2 Grams versus 1 Gram Daily for Gram-Negative Bacteremia

**DOI:** 10.1093/ofid/ofad500.273

**Published:** 2023-11-27

**Authors:** Ricky Huynh-Phan, Andrei Zidaru, Kady Phe

**Affiliations:** Baylor St. Luke's Medical Center, Houston, Texas; Baylor St. Luke's College of Medicine, Houston, Texas; Baylor St. Luke's Medical Center, Houston, Texas

## Abstract

**Background:**

Ceftriaxone (CRO) is commonly utilized for the treatment of Gram-negative infections. However, there are limited data on the appropriate dose of CRO for the treatment of Gram-negative bacteremia. This study aimed to assess the clinical outcomes of CRO 2 grams versus 1 gram daily for Gram-negative bacteremia.

**Methods:**

This was a retrospective, single-center study of patients with Gram-negative bacteremia that received CRO for ≥ 72 hours or over 50% of treatment duration as definitive treatment. Patients were excluded if they had polymicrobial bacteremia, had bacteremia with a Gram-negative organism that was resistant to CRO, or received empiric antibiotics other than CRO for ≥ 72 hours. Definitive treatment was defined as antibiotic utilized after the antibiotic susceptibility report was available to the clinician. Treatment failure was defined as antibiotic escalation due to clinical worsening at any time during CRO therapy. The primary endpoint for this study was 30-day hospital mortality. Secondary endpoints were treatment failure, readmission rates, and adverse reactions.

**Results:**

Of 127 patients included, 57% received CRO 2 grams and 43% received 1 gram. The mean APACHE-II score did not differ between the 2 grams and 1 gram groups (13.8 ± 5.97 versus 12.5 ± 5.61, respectively; p = 0.238). Hypoalbuminemia was present in 21% of patients in the 2 grams group compared to 20% in the 1 gram group (p = 1.00). The most common Gram-negative bacteria isolated were *Escherichia coli* and *Klebsiella* species in 68% and 24% of patients, respectively. Hospital mortality in the entire cohort was 4% (n = 127). Patients receiving 2 grams had a numerically higher 30-day hospital mortality rate (7% versus 0%; p = 0.069). Patients receiving 2 grams also had numerically higher rates of treatment failure (15% versus 5%; p = 0.094). Readmission due to infection did not differ between groups (8% versus 5%; p = 0.731). Overall, ceftriaxone was well tolerated in both groups with no reported adverse reactions.

**Conclusion:**

There was no significant difference in hospital mortality among patients treated with ceftriaxone 2 grams versus 1 gram for Gram-negative bacteremia.

**Disclosures:**

**All Authors**: No reported disclosures

